# Association between gait features assessed by artificial intelligent system and cognitive function decline in patients with silent cerebrovascular disease: study protocol of a multicenter prospective cohort study (ACCURATE-2)

**DOI:** 10.1186/s12883-022-02767-2

**Published:** 2022-06-30

**Authors:** Yan-min Tang, Bei-ni Fei, Xin Li, Jin Zhao, Wei Zhang, Guo-you Qin, Min Hu, Jing Ding, Xin Wang

**Affiliations:** 1 Department of Neurology, Zhongshan Hospital, Fudan University, 180 Fenglin Road, Shanghai, 200032 China; 2 Department of Health Economics, School of Public Health, Fudan University, 130 Dongan Road, Shanghai, 200032 China; 3 Department of Biostatistics, School of Public Health, Fudan University, 130 Dongan Road, Shanghai, 200032 China; 4 Chinese Academy of Sciences Center for Excellence in Brain Science and Intelligence Technology, Chinese Academy of Sciences, 320 Yueyang Road, Shanghai, 200031 China; 5 Department of The State Key Laboratory of Medical Neurobiology and MOE Frontiers Center for Brain Science, Institutes of Brain Science, Fudan University, 131 Dongan Road, Shanghai, 200032 China

**Keywords:** Artificial intelligence, Cognition, Gait features, Microbleeds, Silent brain infarcts, Silent cerebrovascular disease, White matter hyperintensity

## Abstract

**Background:**

Gait disturbances may appear prior to cognitive dysfunction in the early stage of silent cerebrovascular disease (SCD). Subtle changes in gait characteristics may provide an early warning of later cognitive decline. Our team has proposed a vision-based artificial intelligent gait analyzer for the rapid detection of spatiotemporal parameters and walking pattern based on videos of the Timed Up and Go (TUG) test. The primary objective of this study is to investigate the relationship between gait features assessed by our artificial intelligent gait analyzer and cognitive function changes in patients with SCD.

**Methods:**

This will be a multicenter prospective cohort study involving a total of 14 hospitals from Shanghai and Guizhou. One thousand and six hundred patients with SCD aged 60–85 years will be consecutively recruited. Eligible patients will undergo the intelligent gait assessment and neuropsychological evaluation at baseline and at 1-year follow-up. The intelligent gait analyzer will divide participant into normal gait group and abnormal gait group according to their walking performance in the TUG videos at baseline. All participants will be naturally observed during 1-year follow-up period. Primary outcome are the changes in Mini-Mental State Examination (MMSE) score. Secondary outcomes include the changes in intelligent gait spatiotemporal parameters (step length, gait speed, step frequency, step width, standing up time, and turning back time), the changes in scores on other neuropsychological tests (Montreal Cognitive Assessment, the Stroop Color Word Test, and Digit Span Test), falls events, and cerebrovascular events. We hypothesize that both groups will show a decline in MMSE score, but the decrease of MMSE score in the abnormal gait group will be more significant.

**Conclusion:**

This study will be the first to explore the relationship between gait features assessed by an artificial intelligent gait analyzer and cognitive decline in patients with SCD. It will demonstrate whether subtle gait abnormalities detected by the artificial intelligent gait analyzer can act as a cognitive-related marker for patients with SCD.

**Trial registration:**

This trial was registered at ClinicalTrials.gov (NCT04456348; 2 July 2020).

## Background

Silent cerebrovascular disease (SCD) is the most frequently encountered incidental finding on brain imaging in the elderly [1]. According to previous population-based studies, the overall prevalence of SCD ranges from 8 to 28% in the general elderly population and increases with age [2]. Silent lacunar infarctions were detected in 19.9% of community-dwelling people in China who underwent brain computed tomography (CT)/magnetic resonance imaging (MRI) [3]. A large-scale community-based study in Japan reported a 12% prevalence of autopsy-confirmed silent cerebral infarction [4]. Cerebral microinfarcts were reportedly found in 33% of cognitively normal adults at autopsy in longitudinal studies of brain aging [5]. SCD differs from symptomatic cerebral infarcts in its lack of acute stroke-like nervous function defects; however, subtle deficits in motor, cognitive, mental, and daily living activities are often ignored [2]. The proportions of gait apraxia and progressive cognitive decline in patients with SCD are 27.8 and 38.1%, respectively [6]. Bäzner et al. [7] reported that, compared with healthy controls, the patients with SCD showed a slower cadence, shorter step length, and longer double support phase detected by sensors that worsened progressively after a mean 26 months of follow-up. The Framingham Heart Study [8] showed that individuals with large white matter hyperintensity (WMH) volume performed significantly worse in cognitive domains associated with frontal and medial temporal areas compared with those with less or no WMH volumes in a community-based population of non-demented individuals.

SCD is an independent contributing factor to vascular cognitive impairment and dementia [8] that places a heavy burden on patients, their families, and society. The total payments in the US for people aged 65 and older with dementia were estimated to be $305 billion in 2020 and included long-term care and hospice services [9]. In China, the annual total cost associated with dementia was estimated to be $248.71 billion in 2020 [10]. The effective screening of SCD-related cognitive decline for preventing progression to dementia is challenging. Most neuropsychological scales are time-consuming, and their results are affected by language and education level. Gait is an indicator with advantages such as repetition and reliability. There is increasing evidence that a slow gait occurs in the early stages of dementia and may precede the decline on cognitive tests [11]. An acceleration in gait speed decline reportedly occurred 12.1 years before the onset of mild cognitive impairment [12]. There are many common causes of both cognitive decline and gait impairment, such as lesions in the cerebral cortex [13] (such as the prefrontal cortex), impaired executive function, cardiovascular diseases, and diabetes [14]. Motoric cognitive risk syndrome (MCR), a predementia syndrome, involves a slow gait and cognitive complaints and has the potential to be an alternative method for predicting the risk of cognitive decline in low- and middle-income countries [15]. A meta-analysis showed that the adjusted hazard ratio for MCR predicted the risk of developing incident cognitive impairment in individuals aged 60 years and older reached 2.0 (95% confidence interval [CI], 1.7–2.4) [11].

The current clinical gait assessment mostly depends on visual observations of clinical professionals, and its results are affected by large subjective factors. Professional gait analyzers, such as instrumented walkways and marker-based motion capture systems, are not used widespread in the clinical setting because of their high cost and low portability. Our team proposed a vision-based artificial intelligent gait analyzer for the rapid detection of gait spatiotemporal parameters and walking pattern classification based on videos of the Timed Up and Go Test (TUG) without the use of body markers [16]. By learning the gait diagnosis of three experts, the area under the curve of our intelligent gait analyzer for screening gait abnormalities was 0.96, with an accuracy of 90.14%.

The primary objective of this study is to investigate the relationship between gait features assessed by our artificial intelligent gait analyzer and cognitive function changes in patients with SCD. We hypothesize that patients with SCD who are diagnosed as abnormal gait performance by our artificial intelligent gait analyzer will have greater cognitive decline over 1-year follow-up.

## Methods/design

### Study design

The ACCURATE-2 is a multicenter prospective cohort study that follows the changes in scores of neuropsychological test in patients with SCD to compare the differences between normal gait group and abnormal gait group, and analyzes the correlation between gait spatiotemporal parameters and the changes of neuropsychological test scores.

### Setting

The trial will be conducted at the neurology departments of general hospitals. Fourteen secondary or tertiary hospitals located in the Shanghai and Guizhou areas will be included in this study. All staff members of the trial trained.

### Participants

In this study, patients with SCD aged 60–85 years will be consecutively recruited and agree to participate for at least 12 months. Neurologists will identify eligible patients by browsing outpatient cranial MRI images. Patients will be informed about the research contents, and those willing to participate will be given a written informed consent form to sign. After recruitment, suitable patients will be selected according to the inclusion and exclusion criteria.

#### Inclusion criteria

1. Aged 60–85 years.
2. Diagnosed with SCD according to the 2016 statement issued by the American Heart Association and the American Stroke Association [17]:

(A) Absence of clinically recognized acute stroke symptoms.
(B) Cranial MRI taken in the past 1 year having shown one of the following (according to STRIVE [18]):
  - Lacunes of presumed vascular origin: defined as a subcortical, round, or ovoid fluid-filled cavity with a diameter of 3–15 mm showing a low central signal and irregular marginal high signal on T2 fluid-attenuated inversion recovery (T2-FLAIR). The central signal is similar to that of cerebrospinal fluid, and the distribution is consistent with the blood supply area of the perforating artery. Fazekas scores should be ≥2 points.
  - WMH of presumed vascular origin: defined as hyperintensity on T2-FLAIR in the white matter area (periventricular or subcortical), the signal of which differs from cerebrospinal fluid. Fazekas scores should be ≥2 points.
  - Microbleed: defined as a small round area devoid of signal with associated blooming on susceptibility weighted imaging or T2-weighted imaging generally 2–10 mm in diameter. Microbleed lesion number should be ≥5.

(3) Conscious and able to finish all neuropsychological test.
(4) Ability to stand and walk independently without waking aids, and complete the intelligent gait assessment.
(5) Sign the informed consent form.

#### Exclusion criteria

1. Definitively diagnosed as demyelination disease, leukodystrophy, space-occupying lesions, or autoimmune encephalitis, etc.
2. Definitively diagnosed as Parkinson’s disease, normal pressure hydrocephalus, inner ear disease, subacute combined degeneration, peripheral neuropathy, osteoarthritis, or lumbar disease, etc.
3. Definitively diagnosed as Alzheimer’s disease, frontotemporal dementia, or Lewy body dementia, etc.
4. Previous history of severe neurological diseases such as cerebral trauma, epilepsy, alcoholic encephalopathy, or myeleterosis, etc.
5. Severe cardiovascular complications and inability to tolerate the assessment.
6. Severe visual impairment, severe hearing impairment, aphasia, severe cognitive dysfunction, severe gait, balance disturbance and inability to complete the cognition scale and intelligent gait assessments.
7. Refusal to participate in the study.
8. Other anomalies not specified that the researchers considered inappropriate for inclusion in this study.

### Assessments

Clinical data such as demographic characteristics, history of present illness, comorbidities, physical examination findings of the nervous system, previous laboratory examination results and imaging findings, and details of falls events in the past year will be collected. The conversations between doctors and patients during data collection will be recorded by voice recorders, and the audio files will only be used for quality control.

#### Intelligent gait analysis (SAIL system)

The vision-based artificial intelligent gait analyzer we proposed (named SAIL system) includes a data acquisition client and a data analysis server. The data acquisition client consists of a laptop and an RGB-depth camera. The computer application we designed can record the entire TUG test by calling the RGB-depth camera, and upload the video files subsequently to the data analysis server via the network. The data analysis server will detect gait spatiotemporal parameters and walking pattern classification for each patient through the built-in intelligent algorithm and transmits the analysis report back to the data acquisition client through the network.

The TUG videos will be recorded in a bright, clean, and tidy indoor square area (at least 3.8 m × 3.8 m) without any obstruction. A armless chair with standard seat height will be placed on one side, and a visible colored line on the floor will mark a distance of 3 m from the seat. The RGB-depth camera on a 1.2-m-high tripod will be placed 3.8 m away in a vertical line from the midpoint of the footpath. The patients will be required to wear well-fitting trousers and a pair of everyday flat shoes, take off the long coat that reaches the hips, put down the items in their hands, and remove their backpacks before the TUG test. Each participant will be asked to sit on the chair first, stand upon hearing the doctor’s instructions, walk straight to the 3-m mark, turn around, return to the chair, and sit down finally. All participants will complete the TUG test twice at their usual comfortable walking pace, and at least a five-minute break will be given between the tests. Participants can practice 1–2 times to familiarize with the walking test requirements.

On the data analysis server, the pose estimation algorithm will be used to identify the human key body surface points in each frame of the TUG video (such as head, shoulder, elbow, wrist, hip, knee, and ankle), and process the data by filtering and double-threshold signal-detection methods to calculate the six gait spatiotemporal parameters, including step length, gait speed, step frequency, step width, standing up time, and turning back time. Step length is defined as the average distance from the landing of one foot to the subsequent landing of the other foot, and a shorter step length will indicate worse gait performance. Gait speed is defined as the average velocity of walking during the straight parts of the TUG test, and a slower gait speed will indicate worse gait performance. Step frequency is defined as the average steps taken per second, and a slower step frequency will indicate worse gait performance. Step width is defined as the depth distance between two heels, and a wider step width will indicate a worse gait balance. Standing up time is defined as the time interval from the beginning of the video to the timepoint when the height of two shoulders reach the highest, which includes reaction times and stand-up action times, and a longer time will indicate worse gait performance. Turning back time is defined as the time between the start and end of the shoulder twisting, and a longer time will indicate worse gait performance. The native Bayes classifier that has learned the expert gold standard before will be applied to classify gait performance as normal or abnormal.

#### Neuropsychological evaluation

Participants will be evaluated on the following neuropsychological tests by trained doctors:

1. Mini-Mental State Examination (MMSE), Chinese version translated by Mingyuan Zhang: evaluates time and place orientation, attention and calculation, recall, language, and copying. Scores range from 0 to 30 points, with a lower score indicating worse cognitive function.
2. Montreal Cognitive Assessment (MoCA), Beijing version: evaluates visual-spatial ability, executive function, naming, memory, attention, language, abstraction, and orientation. Scores range from 0 to 30 points, with a lower score indicating worse cognitive function.
3. The Stroop Color word test (CWT), Huashan version: evaluates semantic activation, dominant response inhibition, attention, working memory, and information processing speed. The time spent in each card and the number of errors will be recorded. A longer time cost or more errors indicate worse cognitive function.
4. The digit span test (DST), a subtest of the Chinese version of the Wechsler Adult Intelligence Scale-Revised: evaluates immediate memory and attention. A shorter string of repeated numbers indicates worse cognitive function.

### Grouping and follow-up plan design

**Fig. 1:**
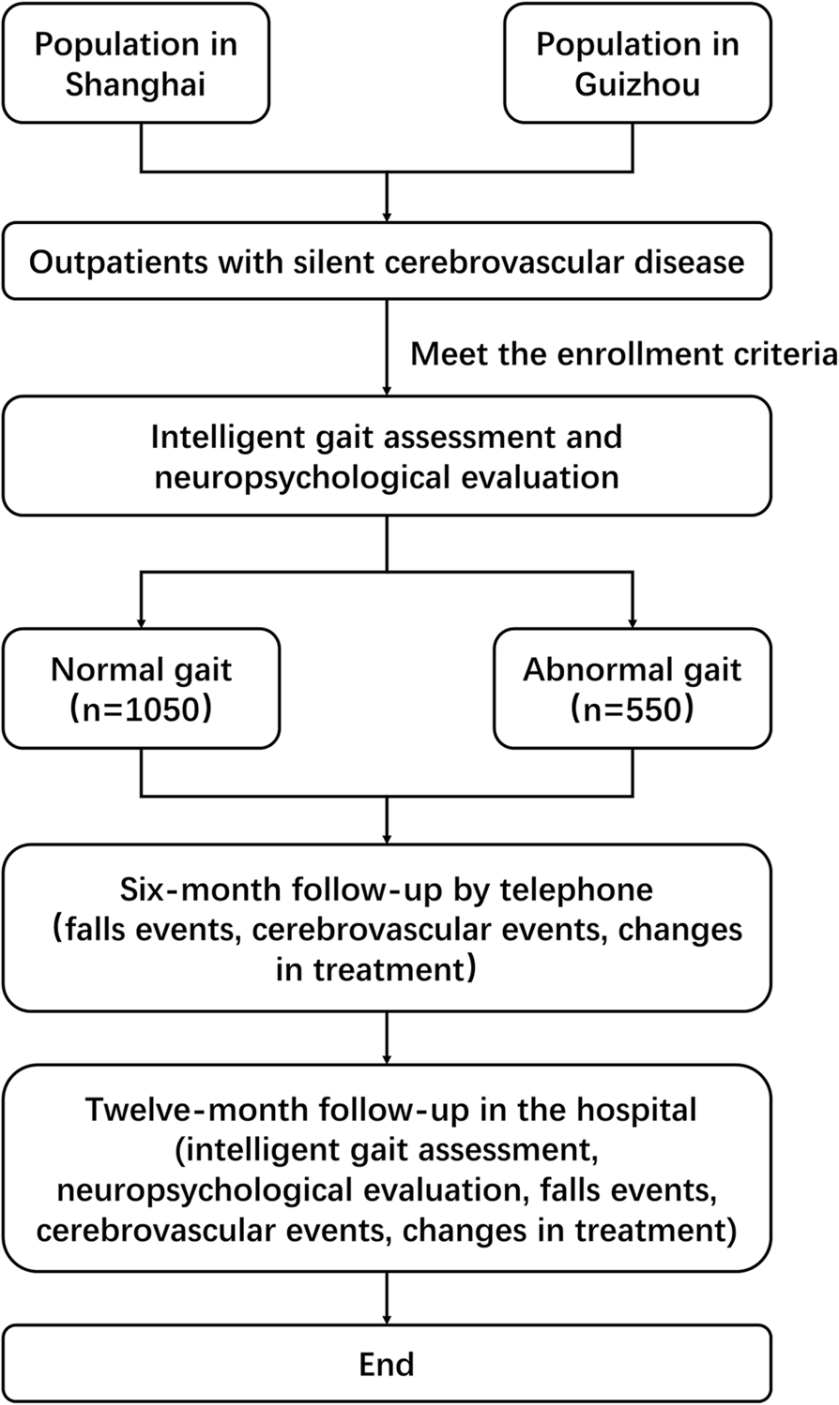
Flow diagram of the ACCURATE-2 study

During the baseline period, all participants will be evaluated by intelligent gait analyzer and neuropsychological tests. According to the intelligent gait report, each patient will be classified as normal gait group or abnormal gait group. All participants will be naturally observed for at least 1 year, and all their medical behaviors will be recorded. At six months after enrollment, all patients will be interviewed by telephone to collect falls events, cerebrovascular events, and the changes in medical behaviors during the follow-up period. The participants will be invited to undergo intelligent gait assessment and neuropsychological evaluation in the hospital at 12 months after enrollment, and falls events, cerebrovascular events, the changes in medical behaviors during the follow-up period will be collected. The details are presented in Fig. 1 and Table 1. All data will be encrypted and stored on the local server in Zhongshan hospital.

**Table 1:** Overview of the study assessments

Assessment	Baseline	Six months	One year
Method	Face to face	Telephone	Face to face
Cranial MRI	×		×
Intelligent gait assessment	×		×
Neuropsychological evaluation			
MMSE	×		×
MoCA	×		×
CWT	×		×
DST	×		×
Falls events	×	×	×
Cerebrovascular events		×	×
Changes in treatment		×	×

× indicates that the assessment took place. *CWT* the Stroop Color Word Test, *DST* the Digit Span Test, *MMSE* Mini-Mental State Examination, *MoCA* Montreal Cognitive Assessment

### Outcomes

The primary outcome of this study will be the changes in the MMSE score in patients with SCD after 1-year follow-up.

The secondary outcomes of this study will be the changes in intelligent gait parameters (step length, gait speed, step frequency, step width, standing up time, and turning back time) and the changes in scores on other neuropsychological tests (MoCA, CWT, and DST) after 1-year follow-up, the prevalence of abnormal gait and cognition decline in patients with SCD, and the incidence of cerebrovascular events and falls in patients with SCD.

### Sample size

Referring to previous studies of cognitive decline among patients with SCD [19–23], we assume that the MMSE score will decrease by 0.5 ± 0.5 points in the normal gait group and 0.7 ± 1.2 points in the abnormal gait group within 1 year; the ratio of abnormal gait to normal gait among outpatients with SCD is 1:2. Test efficiency 1-beta is set at 90%, while test level alpha is set at 0.05. An estimated 20% shedding rate, sample size of 1050 patients with a normal gait and 550 patients with an abnormal gait will be required. Therefore, a total of 1600 patients will be included.

### Statistical analysis

All measurement data will be tested using the Shapiro-Wilk normality test. Normally distributed continuous measurement data will be expressed as mean and standard deviation, while non-normally distributed measurement data will be expressed as median and interquartile range. The t-test, Wilcoxon signed-ranks test, repeated measures t-test, or linear generalized model will be used to compare measurement data. The enumeration data will be expressed as a composition ratio or rate. The chi-square test or Fisher exact method will be used to compare enumeration data. Pearson linear correlation analysis or Spearman correlation analysis will be performed for correlation analysis. Survival will be estimated using a Kaplan-Meier survival curve. The propensity score matching method will be used to minimize the influence of other confounders. Subgroup analyses will include region and hospital levels. All cases with missing values will be deleted. A significant difference will be identified *P* < 0.05.

## Discussion

Our study will be the first to explore the relationship between gait features assessed by an artificial intelligent gait analyzer and cognitive decline in patients with SCD. Gait features detected by computer vision technology may provide some clues of subtle gait changes, which has the potential to be a new cognitive-related marker for patients with SCD in the future. Meanwhile, this study will provide clinical evidence for the risk factors of SCD-related gait disorders and cognitive dysfunction and the epidemiology of adverse outcomes in SCD.

SCD is a type of cerebrovascular disease diagnosed according to its neuroimaging features and clinical manifestations [17, 18]. However, it is not cost-effective and feasible to screen asymptomatic elderly people and follow up patients with SCD using routine cranial MRI. Even with symptomatic strokes, approximately 90% of patients in China underwent non-contrast brain CT, while only 50% underwent brain MRI [24]. Therefore, it is very important to develop a specific quantitative screening and evaluation method for the clinical characteristics of patients with SCD. Gait assessments is expected to become a promising evaluation method for patients with SCD: it not only evaluates the degree of gait disorders, but also predicts the risk of long-term cognitive decline and other adverse outcomes. Verghese et al. [25] found that the hazard ratio of vascular dementia for non-dementia individuals with neurological gait abnormalities at baseline was 3.46 (95% CI, 1.86–6.42) after a median 6.6-year follow-up. Dumurgier et al. [26] proposed that a patient’s gait was slow up 7 years prior to dementia, and those with a greater decline in gait speed had a higher risk of developing dementia. Similarly, Montero-Odasso et al. [27] reported that greater changes in dual-task gait speed in patients with mild cognitive impairment were associated with dementia progression. The population-based RUN DMC Study [28] reported that for every 0.1 m/s decrease in gait speed, the hazard ratio of 8-year mortality in patients with SCD was 1.15 (95% CI, 1.06–1.24). Most of the above-mentioned studies use sensors to measure gait parameters, with the disadvantage that the equipment is expensive and difficult to apply in primary hospitals. Future investigations should focus on early screening methods for high-risk population to effectively reduce the burden of dementia, falls, and stroke in SCD. Our intelligent gait analyzer can quantify gait parameters and classify gait pattern with high-precision based on intelligent pose estimation algorithm, which is expected to be further applied in primary hospitals, communities and even families. Moreover, most previous large-scale population-based SCD studies were conducted in developed countries, and the patients were mostly White. Our study includes Chinese multi-ethnic yellow race, which may provide more clues for the early detection and prevention of SCD.

## Data Availability

Data sharing is not applicable to this article as no datasets were generated or analysed during the current study. The final results of this study will be published in peer-reviewed journals.

## Abbreviations

CWT: the Stroop Color Word Test
DST: Digit Span Test
MCR: Motoric Cognitive Risk Syndrome
MMSE: Mini-Mental State Examination
MoCA: Montreal Cognitive Assessment
MRI: Magnetic Resonance Imaging
SCD: Silent Cerebrovascular Disease
STRIVE: the STandards for ReportIng Vascular changes on Euroimaging
T2-FLAIR: T2 fluid-attenuated inversion recovery
TUG: Timed Up and Go
WMH: White Matter Hyperintensity
